# Growth From Birth to Adulthood and Bone Phenotype in Early Old Age: A British Birth Cohort Study

**DOI:** 10.1002/jbmr.2008

**Published:** 2013-12-19

**Authors:** Diana Kuh, Andrew K Wills, Imran Shah, Ann Prentice, Rebecca Hardy, Judith E Adams, Kate Ward, Cyrus Cooper, National Survey for Health and Development (NSHD) Scientific and Data Collection Team

**Affiliations:** 1 Medical Research Council (MRC) Unit for Lifelong Health and Ageing, Institute of Epidemiology and Health Care, University College LondonLondon, UK; 2 MRC Centre for Causal Analysis in Translational Epidemiology, School of Social and Community Medicine, University of BristolBristol, UK; 3 MRC Human Nutrition ResearchCambridge, UK; 4 Clinical Radiology and Manchester Academic Health Service Centre, The Royal InfirmaryManchester, UK; 5 MRC Lifecourse Epidemiology Unit, University of SouthamptonSouthampton, UK

**Keywords:** cohort, growth trajectory, bone strength, bone area, BMD

## Abstract

There is growing evidence that early growth influences bone mass in later life but most studies are limited to birth weight and/or early infant growth and dual-energy X-ray absorptiometry (DXA) measurements. In a British birth cohort study with prospective measures of lifetime height and weight, we investigated the growth trajectory in relation to bone in males (M) and females (F) at 60 to 64 years old. Outcomes were DXA measures of hip and spine areal bone density (aBMD) (*n* = 1658) and pQCT measures of distal and diaphyseal radius cross-sectional area (CSA), strength, and volumetric bone density (vBMD) (*n* = 1350 of the 1658). Regression models examined percentage change in bone parameters with standardized measures of birth weight, height, and weight. A series of conditional growth models were fitted for height and weight gain (using intervals: birth–2, 2–4, 4–7, 7–15, 15–20, 20–36, and 36–64 years) and height gain (using intervals: 2–4, 4–7, 7–15, and 15–36 years). Birth weight was positively related to bone CSA (M: 1.4%; 95% confidence interval [CI], 0.3%–2.5%; F: 1.3%; 95% CI, 0.3%–2.4% per 1 SD increase in birth weight for diaphyseal CSA) and strength (M: 1.8%; 95% CI, 0.3–3.4; F: 2.0%; 95% CI, 0.5–3.5). No positive associations were found with trabecular, total, or cortical vBMD. One SD change in prepubertal and postpubertal height and weight velocities were associated with between 2% and 5% greater bone CSA and strength. Height gain in later years was negatively associated with trabecular vBMD. Weight gain velocity during the adult years was positively associated with up to 4% greater trabecular and total BMD, and 4% greater aBMD at hip and spine. In a cohort born in the early post-war period, higher birth weight, gaining weight and height faster than others, particularly through the prepubertal and postpubertal periods, was positively related to bone strength, mostly through greater bone CSA, at 60 to 64 years.

## Introduction

Growth during the intrauterine and early-postnatal period is associated with future bone phenotype and fracture risk.^1^–^8^ During this time, rapid mineral gain and the relatively plastic skeleton make genome and early environmental interactions quite likely. The majority of studies investigating childhood growth and adult bone health and fracture risk have focused on associations between birth weight or weight at 1 year old and bone phenotype. Fewer studies have examined the entire growth trajectory in relation to adult bone mass, including the prepubertal, pubertal, and postpubertal periods.

Recent systematic reviews have shown that prenatal growth, as indexed by birth weight, is associated with bone area (BA) and bone mineral content (BMC) but not generally with areal bone mineral density (aBMD) during adulthood,^1^,^7^ suggesting that the effect of birth weight on bone mass is largely mediated through its relationship with adult bone size (length and cross-sectional area [CSA]). These findings held for a number of sites, including the lumbar spine and hip. In the Hertfordshire cohort study, birth weight and weight gain in the first year of life were associated with BMC and BA but not aBMD assessed in the seventh decade, after accounting for adult weight.^3^ More recently, in the New Delhi birth cohort followed to age 39 years,^8^ greater skeletal growth and body mass index (BMI) gain in utero and infancy were associated with higher peak BMC, and greater BMI gain in childhood and adolescence were associated with peak aBMD; these associations were explained by adult height and BMI. In two large studies of men and women born in Helsinki University Central Hospital during 1924 to 1933 or 1934 to 1944, hip fracture risk was shown to be associated with poor rate of childhood height and weight gain from age 7 to 15 years^2^ or low BMI gain between 1 and 12 years^5^ but current body size was not available in these studies.

The attenuation of the relationships between birth weight, and weight at 1 year and later bone phenotype,^4^,^6^,^9^ after adjustment for body and/or bone size indicates that the majority of the influence is on the size of the skeletal envelope rather than mineralization. Supportive of this are observations from studies using peripheral quantitative computed tomography (pQCT), which reported that birth weight was not associated with radial or tibial volumetric bone mineral density (vBMD), but was associated with CSA and the stress-strain index (SSI),^6^ an in vivo estimate of bone strength.^4^,^6^,^9^ Because SSI is calculated using the size, density, and distribution of the bone,^10^ these data again suggest the effects of birth weight are mediated through skeletal size not bone mineralization.

To our knowledge there are no studies that have collected weight and height measurements from birth across life and measured the bone phenotype in early old age. We have recently completed bone measurements at 60 to 64 years old in the Medical Research Council (MRC) National Survey for Health and Development (NSHD), a birth cohort study. The aim of the current study was to use prospective measures of birth weight, height, and weight across life, and cross-sectional dual-energy X-ray absorptiometry (DXA) and pQCT measurements in early old age to examine the associations between the growth trajectory at different stages in childhood and pQCT measures of diaphysis and medullary CSA, SSI, and cortical vBMD of the diaphyseal radius, distal CSA, total vBMD, and trabecular vBMD at the distal radius and with DXA measures of aBMD at the total hip and lumbar spine.

## Subjects and Methods

The MRC NSHD is based on a nationally representative sample of 5362 births out of all the single, legitimate births that occurred in 1 week in March 1946 in England, Scotland, and Wales. The whole sample has been followed up 23 times and the latest data collection took place when participants were 60 to 64 years old (between 2006 and 2010).^11^ Study members still alive and with a known current address in England, Scotland, or Wales were invited for an assessment at one of six clinical research facilities (CRFs); those unable or unwilling to travel were offered a home visit by a research nurse. Invitations were not sent to those who had died (*n* = 778), were living abroad (*n* = 570), had previously withdrawn from the study (*n* = 594), or had been lost to follow-up (*n* = 564). Of the 2856 invited, 2229 (78%) were assessed: 1690 (59%) attended a CRF and the remaining 539 were visited at home. The participating sample remains broadly representative of native born British men and women of the same age.^12^

### Musculoskeletal assessment

Of the 1690 attending one of the six CRFs, 1658 had a DXA bone scan; of these, 1350 also had a pQCT scan of the nondominant radius (distal 4% and diaphyseal 50% sites) at one of five CRFs where this equipment was provided. All the sites used QDR 4500 Discovery (Hologic Inc., Bedford, MA, USA) and XCT 2000 (Stratec, Pforzheim, Germany) DXA and pQCT scanners, respectively. For consistency and optimization in scan acquisition, a detailed training protocol booklet and illustrative CD were prepared, for each center, on subject and phantom scanning. For cross calibration the European Spine Phantom [ESP]; number 04–220^13^), which has three trabecular BMD value (50, 100, 200 mg/cm^3^) inserts, was scanned at each center at the start and end of the study period. The “known” BMD values of each ESP vertebral body were plotted against the “measured” BMD values and coefficients from the line of best fit were recorded^14^ and used to calculate standardized BMD (sBMD). The European forearm phantom (EFP)^14^ similarly was scanned 10 times on each pQCT scanner at the beginning and end of the study in each center and differences between scanners were tested for total and trabecular vBMD and total area of section 1 to 3; cortical vBMD was tested for section 4 only. No cross-calibration was necessary for pQCT measurements.^14^ For quality assurance (QA) the phantoms provided by the scanner manufacturer were scanned daily and the results were sent to the bone coordinating center monthly for scrutiny. All data were centrally analyzed and scrutinized by author JEA’s laboratory.

The pQCT scans at the distal 4% site provided measures of trabecular and total vBMD and distal CSA, and at the 50% site provided CSA of the diaphysis and the medullary cavity (medullary CSA), and cortical vBMD and polar SSI (mm^3^)^15^ were extracted.

The DXA measures included in this analysis were aBMD for the lumbar spine (L_1_–L_4_) and total hip. Information on prescribed oral glucocorticoids, aromatase inhibitors, and all medications taken for osteoporosis was obtained.

### Body size and velocity measurements

Birth weight (kg), height (m), and weight (kg) in childhood (at 2, 4, 6, 7, and 15 years), and in adult life (at 20, 26, 36, 43, 53, and 60 to 64 years) were measured using standardized protocols, except at ages 20 and 26 when they were self-reported. Because the first two adult measurements of height were self-reported we used measured height at 36 years as final achieved height. We derived weight gain velocities (from birth to 2, 2 to 4, 4 to 7, 7 to 15, 15 to 20, 20 to 36, and 36 to 60–64 years), and height gain velocities (from 2 to 4, 4 to 7, 7 to 15, and 15 to 36 years) by subtracting the measurement at the younger age from the measurement at the older age, and dividing by the difference in those ages, measured in months since birth. We then standardized these velocities, and the original heights and weights, to give a mean of 0 and an SD = 1.

### Statistical analysis

Stata v10.1 (StatCorp, College Station, TX, USA) was used for all analyses. Means and SDs for all the bone outcomes were derived. Regression models used natural logarithms for all bone variables. We fitted separate models for men and women but tests for sex interactions were carried out in models using both sexes. The coefficients from these models are presented as the percentage difference in the bone parameter at 60 to 64 years for a 1 SD higher *Z*-score for weight or height at the relevant age.

#### Analyses of the growth trajectory and DXA and pQCT outcomes at 60 to 64 years

First, we used regression models to investigate the relationship between the transformed birth weight, weight and height at each age, and the adult bone outcomes using all available data, first adjusted only for age at measurement and then mutually adjusting for height and weight (except for birth weight because birth length was not available). We present mutually adjusted estimates as plots for visual inspection in Supplementary Figs. 1 to 6 (regression estimates available on request). Nonlinearity in the relationship between the *Z*-scores at each age and each bone outcome was assessed using a quadratic term. This was followed up by visually inspecting scatter plots, where significant quadratic terms were found. Linear relationships were ultimately deemed sufficient in all cases.

Second, we used conditional growth models to examine the associations of the growth trajectory with adult bone outcomes. A series of conditional growth models for each growth interval were fitted, which included the following transformed variables: height and weight velocities for that interval, and height and weight at the beginning of the interval. The coefficients for the velocity variables from these models are presented as the percentage difference in the bone parameter for a 1 SD increase in the *Z*-scores for weight or height velocity independent of earlier growth. They offer a prospective view of the net association between each growth interval and later bone outcome, in path model terminology; ie, the sum of the direct (independent) effect and indirect effects acting through future size. We present the estimates as plots for visual inspection and as Supplementary Tables 1 to 5.

## Results

The median and interquartile range for the pQCT-derived and DXA-derived bone outcomes, and for the measures of height and weight across life, showed expected sex differences (Table 1). Compared with women, men were taller and heavier with greater diaphysis and medullary CSA, and SSI at the 50% radius site, and distal CSA, total and trabecular vBMD at the 4% radius site; areal BMD was greater at hip and spine. There were 29 participants taking prescribed oral glucocorticoids, aromatase inhibitors, or medications for osteoporosis. There were no significant differences in height, weight, or DXA measurement between those with a pQCT and a DXA measure, compared to those with only a DXA measure. Men and women who had a DXA or pQCT scan at the clinic visit were, on average, 2 cm taller (*p* < 0.001) and the women were 1.9 kg lighter (*p* = 0.05) than those who had a home visit and did not have bone scans.

**Table 1:** Median and IQR for pQCT-Derived and DXA-Derived Outcomes at 60 to 64 Years Old and Height and Weight at Various Ages

	Men			Women		
	*n*	Median	IQR	*n*	Median	IQR
pQCT measures						
pQCT cortical sites						
50% radius						
Diaphysis CSA (mm^2^)	657	152.0	139.5, 168.1	697	110.9	102.2, 121.0
Medullary CSA (mm^2^)	657	41.0	33.5, 50.0	697	33.1	27.0, 41.7
Polar stress strain index (mm^3^)	658	343.8	296.0, 390.1	697	207.4	178.9, 236.4
pQCT trabecular sites						
Distal radius (4%)						
Distal CSA (mm^2^)	657	376.2	333.0, 424.5	688	291.4	259.7, 326.5
pQCT 50% radius						
Cortical vBMD (mg/cm^3^)	658	1161.9	1137.8, 1182.3	697	1153.3	1123.7, 1175.5
pQCT distal radius (4%)						
Trabecular vBMD (mg/cm^3^)	657	203.6	177.4, 232.7	688	169.4	144.4, 199.4
Total density vBMD (mg/cm^3^)	657	385.5	342.6, 434.2	688	318.7	281.8, 376.0
DXA measures						
DXA aBMD (g/cm^2^)						
Spine L_1_–L_4_ aBMD	790	1.03	0.92, 1.17	861	0.92	0.83, 1.05
Total hip aBMD	780	0.99	0.90, 1.10	856	0.86	0.78, 0.95
Height measurements (m)						
Age (years) at assessment						
2	646	0.86	0.84, 0.89	693	0.86	0.81, 0.86
4	701	1.04	1.02, 1.07	763	1.04	0.99, 1.07
6	669	1.14	1.12, 1.19	723	1.14	1.12, 1.17
7	684	1.22	1.17, 1.24	753	1.19	1.17, 1.24
11	670	1.42	1.37, 1.45	732	1.42	1.37, 1.47
15	627	1.63	1.57, 1.70	676	1.60	1.55, 1.63
20	649	1.78	1.73, 1.80	739	1.63	1.57, 1.68
26	690	1.78	1.73, 1.80	771	1.63	1.57, 1.68
36	713	1.76	1.72, 1.80	791	1.63	1.59, 1.67
60–64	792	1.75	1.71, 1.79	866	1.62	1.58, 1.66
Weight measurements (kg)						
Age (years) at assessment						
Birth	791	3.41	3.18, 3.75	862	3.30	3.07, 3.64
2	666	13.20	12.7, 14.1	726	12.70	11.8, 13.6
4	723	17.20	15.9, 19.1	780	16.80	15.9, 18.6
6	674	20.90	19.1, 22.7	731	20.40	18.6, 22.2
7	668	23.10	21.1, 24.9	727	22.20	20.9, 24.5
11	672	33.80	30.4, 37.2	726	34.00	30.4, 39.0
15	628	51.70	45.4, 57.6	675	51.30	47.2, 56.7
20	652	69.90	64.4, 76.2	734	57.20	52.6, 61.7
26	690	71.70	66.7, 79.4	770	57.20	53.5, 63.5
36	714	75.00	68.5, 82.0	792	60.00	55.0, 66.0
43	743	77.20	70.6, 85.0	823	63.40	58.0, 70.4
53	740	81.80	74.9, 90.4	835	68.20	61.4, 77.5
60–64	792	84	76.4, 93.0	866	69.80	62.8, 80.2

IQR = interquartile range; DXA = dual-energy X-ray absorptiometry; CSA = cross-sectional area; vBMD = volumetric bone mineral density; aBMD = areal bone mineral density.

### Growth to adulthood and radius diaphysis, distal and medullary CSA, and strength at 60 to 64 years

Cross-sectional bone area (diaphyseal, medullary, and distal) and SSI measures were positively associated with birth weight (Table 2). These estimates were attenuated after adjustment for current height and weight. The adjusted estimates were strongest for diaphysis CSA and SSI; for example, with SSI there was a 2.0% (95% confidence interval [CI], 0.5% to 3.5%) increase in SSI per 1 SD increase in birth weight in women. However no evidence of an association between birth weight and medullary CSA remained after adjustment for current height and weight.

**Table 2:** Percentage Difference in Bone Outcomes at 60 to 64 Years Old for a 1 SD Increase in Birth Weight

		Men				Women			
	*n*	% Difference (95% CI), unadjusted	*p*	% Difference (95% CI), adjusted for current height and weight	*p*	% Difference (95% CI), unadjusted	*p*	% Difference (95% CI), adjusted for current height and weight	*p*
pQCT diaphyseal radius (50%) site									
50% radius									
Diaphysis CSA (mm^2^)	1349	2.90 (1.76, 4.05)	<0.001	1.43 (0.33, 2.53)	0.011	2.68 (1.55, 3.82)	<0.001	1.33 (0.27, 2.40)	0.014
Medullary CSA (mm^2^)	1348	3.77 (1.04, 6.50)	0.007	1.79 (–0.99, 4.57)	0.206	3.09 (0.22, 5.96)	0.035	1.24 (–1.63, 4.11)	0.395
Polar stress strain index (mm^3^)	1350	3.94 (2.31, 5.57)	<0.001	1.83 (0.26, 3.40)	0.022	3.78 (2.19, 5.36)	<0.001	1.99 (0.51, 3.49)	0.009
pQCT distal radius (4%) site									
Distal CSA (mm^2^)	1340	2.62 (0.94, 4.29)	0.002	1.55 (–0.16, 3.26)	0.076	2.89 (1.39, 4.38)	<0.001	1.38 (–0.06, 2.82)	0.061
pQCT diaphyseal radius (50%) site									
Cortical vBMD (mg/cm^3^)	1350	–0.02 (–0.26, 0.22)	0.865	–0.01 (–0.26, 0.24)	0.932	–0.18 (–0.46, 0.09)	0.196	–0.20 (–0.48, 0.08)	0.166
pQCT distal radius (4%) site									
Trabecular vBMD (mg/cm^3^)	1340	–0.81 (–2.55, 0.93)	0.359	–0.64 (–2.44, 1.15)	0.481	–1.78 (–3.88, 0.33)	0.098	–1.36 (–3.49, 0.77)	0.211
Total vBMD (mg/cm^3^)	1340	–1.49 (–2.87, –0.11)	0.035	–1.46 (–2.90, –0.03)	0.045	–1.31 (–3.04, 0.42)	0.137	–0.88 (–2.62, 0.86)	0.322
DXA aBMD(g/cm^2^)									
Spine L_1_–L_4_ aBMD (g/cm^2^)	1646	1.17 (–0.13, 2.47)	0.077	0.19 (–1.09, 1.47)	0.773	–0.18 (–1.44, 1.07)	0.774	–0.82 (–2.04, 0.40)	0.188
Hip aBMD (g/cm^2^)	1631	0.99 (–0.11, 2.08)	0.077	–0.07 (–1.07, 0.94)	0.895	0.22 (–0.89, 1.32)	0.698	–0.47 (–1.44, 0.50)	0.340
SA-BMC(g/cm^2^)									
Spine	1646	0.34 (–0.77, 1.45)	0.552	0.20 (–0.89, 1.28)	0.719	–0.60 (–1.73, 0.53)	0.297	–0.83 (–1.93, 0.26)	0.137
Hip	1631	0.54 (–0.48, 1.57)	0.297	0.12 (–0.78, 1.03)	0.787	–0.08 (1.11, 0.94)	0.873	–0.39 (–1.28, 0.50)	0.387

Unadjusted and adjusted for standardized height and weight at 60 to 64 years of age.

CI = confidence interval; CSA = cross-sectional area; vBMD = volumetric bone mineral density; DXA = dual-energy X-ray absorptiometry; aBMD = areal bone mineral density; SA-BMC = size-adjusted bone mineral content.

**Figure 1:**
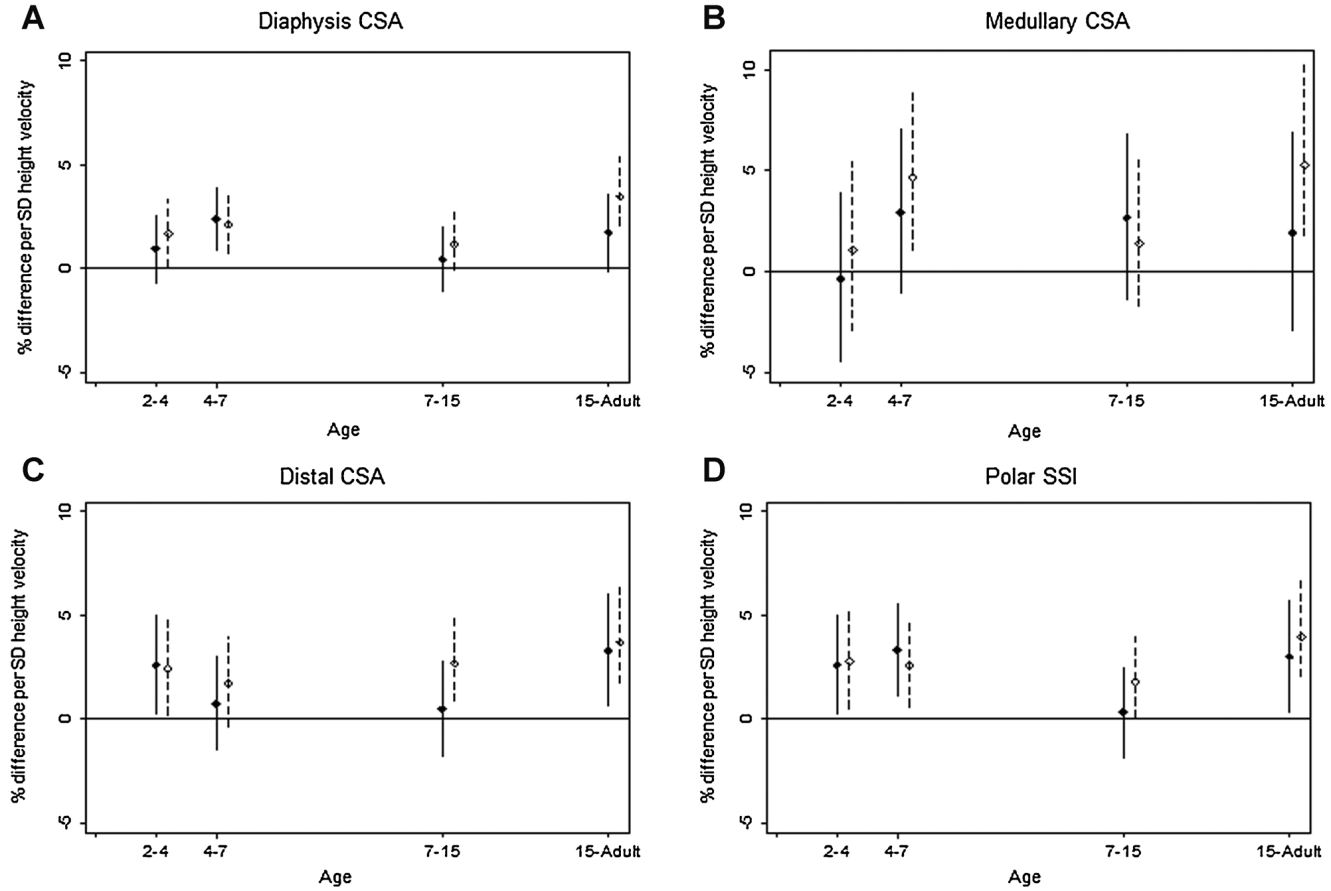
Height velocity. Percentage difference and 95% CI in (*A*) radius diaphysis CSA, (*B*) medullary CSA, and (*C*) polar SSI (50% site), and (*D*) distal CSA (4% site), at 60 to 64 years per 1 SD increase in height velocity, adjusted for standardized height and weight at the beginning of the growth interval, and corresponding standardized weight velocity. Solid line for males; dashed line for females.

**Figure 2:**
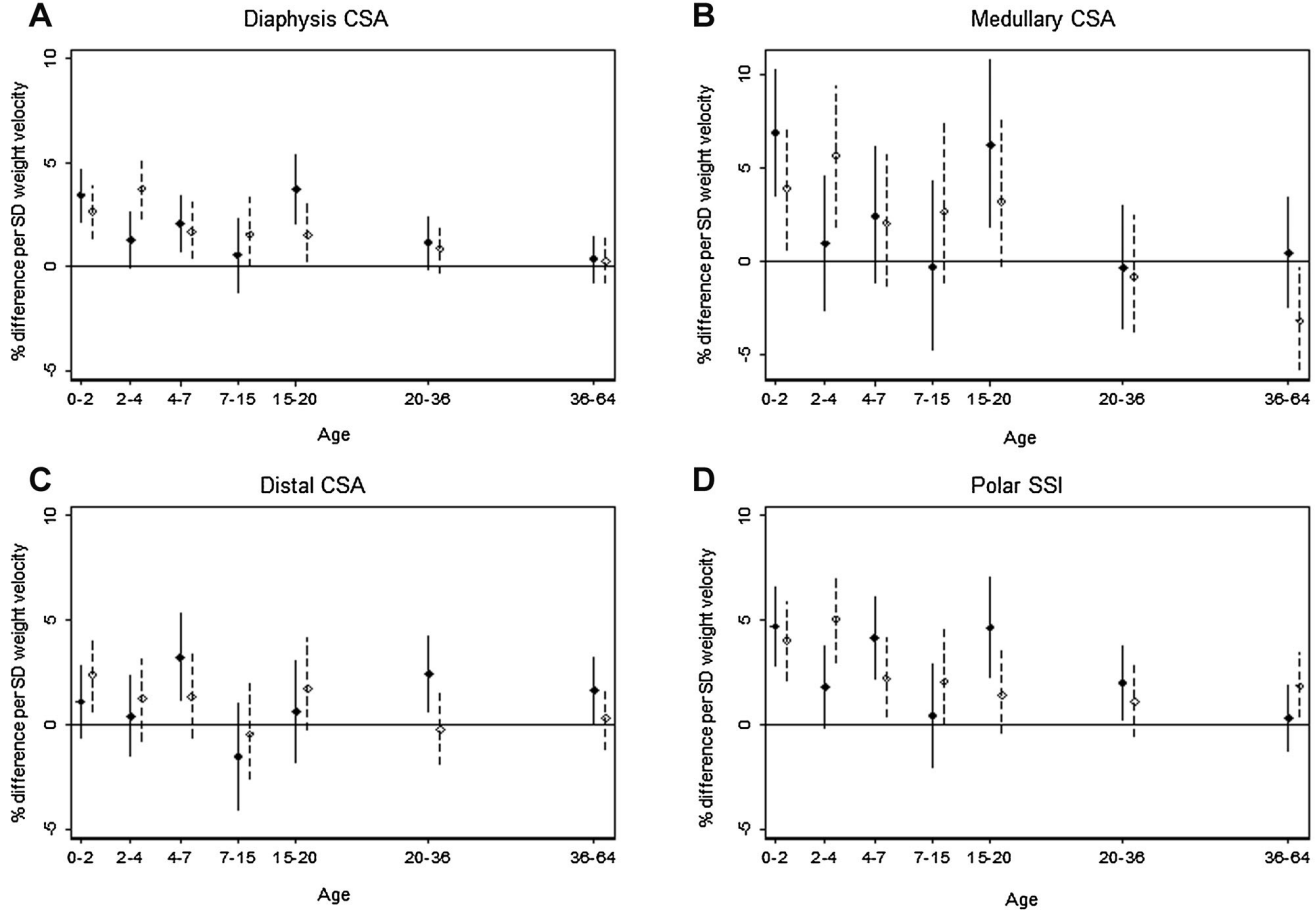
Weight velocity. Percentage difference, and 95% CI in (*A*) radius diaphysis CSA and (*B*) medullary CSA (50% site), and (*C*) polar SSI and (*D*) distal CSA (4% site), at 60 to 64 years per 1 SD increase in weight velocity, adjusted for standardized height and weight at the beginning of the growth interval and corresponding standardized height velocity. Solid line for males; dashed line for females.

Results from the conditional growth models (that included height and weight velocities for a given growth interval, and height and weight at the beginning of that interval are presented in Figs. 1 and 2 and Supplementary Tables 1 and 2). They show positive associations between prepubertal height gain (up to 7 years) and adult bone CSA and strength. For diaphysis and medullary CSA (Fig. 1*A*, *B*), the estimates were greatest for height gain 4 to 7 years; for example, in women, medullary CSA increased by 4.7% (95% CI, 1.0% to 8.9%) for a 1 SD increase in height velocity. For distal CSA (Fig. 1*C*), the estimates were greatest for height gain in the years 2 to 4. For SSI (Fig. 1*D*) there were associations between height gain in 2 to 4 and 4 to 7 years and the estimates were of similar size. In the pubertal (7–15 years) and postpubertal (15–adult) periods, the latter estimates were strongest for all four measures except for medullary CSA in men. For example, medullary CSA in women increased by 5.3% (95% CI, 1.7% to 10.5%) for a 1 SD increase in height velocity between 15 and 36 years.

Weight gain up to age 7 years and from 15 to 20 years was more strongly positively associated with bone CSA at either the 4% or 50% site and SSI than that during 7 to 15 years or in adulthood (Fig. 2, Supplementary Table 2). Estimates were strongest for diaphysis (Fig. 2*A*) and medullary (Fig. 2*B*) CSA and SSI (Fig. 2*D*) for 1 SD of weight gain velocity at 0 to 2 and 15 to 20 years in men, and at 2 to 4 years in women; the estimate for medullary CSA in women was negative for weight gain between 36 and 64 years. The strongest estimates for the distal CSA (Fig. 2*C*) were for a 1 SD increase in weight velocity at 4 to 7 years (3.2%; 95% CI, 1.1% to 5.3%) and 20 to 36 years (2.4%; 95% CI, 0.6% to 4.3%) for men, and at 0 to 2 years for women (2.4%; 95% CI, 0.6% to 4.2%).

### Growth to adulthood and pQCT measures of radius trabecular, total, and cortical vBMD at 60 to 64 years

Birth weight was not associated with vBMD at either the diaphyseal or distal radius (Table 2) with one exception: total vBMD at the distal radius in males was inversely associated with birth weight before and after adjusting for current weight and height.

**Figure 3:**
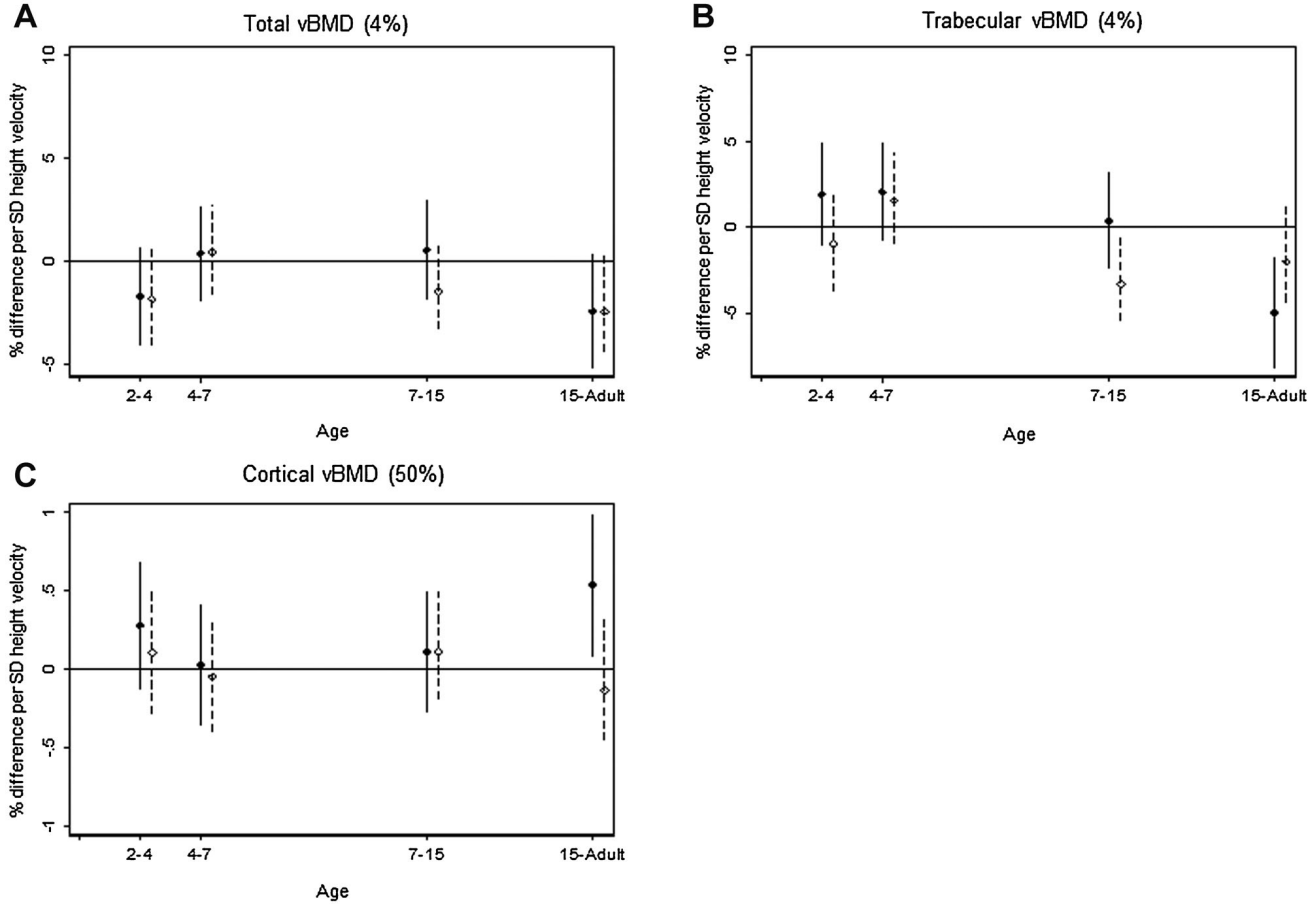
Height velocity. Percentage difference and 95% CI in (*A*) total vBMD and (*B*) trabecular vBMD (4% site), and (*C*) cortical vBMD (50% site), at 60 to 64 years per 1 SD increase in height velocity, adjusted for standardized height and weight at the beginning of the growth interval and corresponding standardized weight velocity. Solid line for males; dashed line for females.

**Figure 4:**
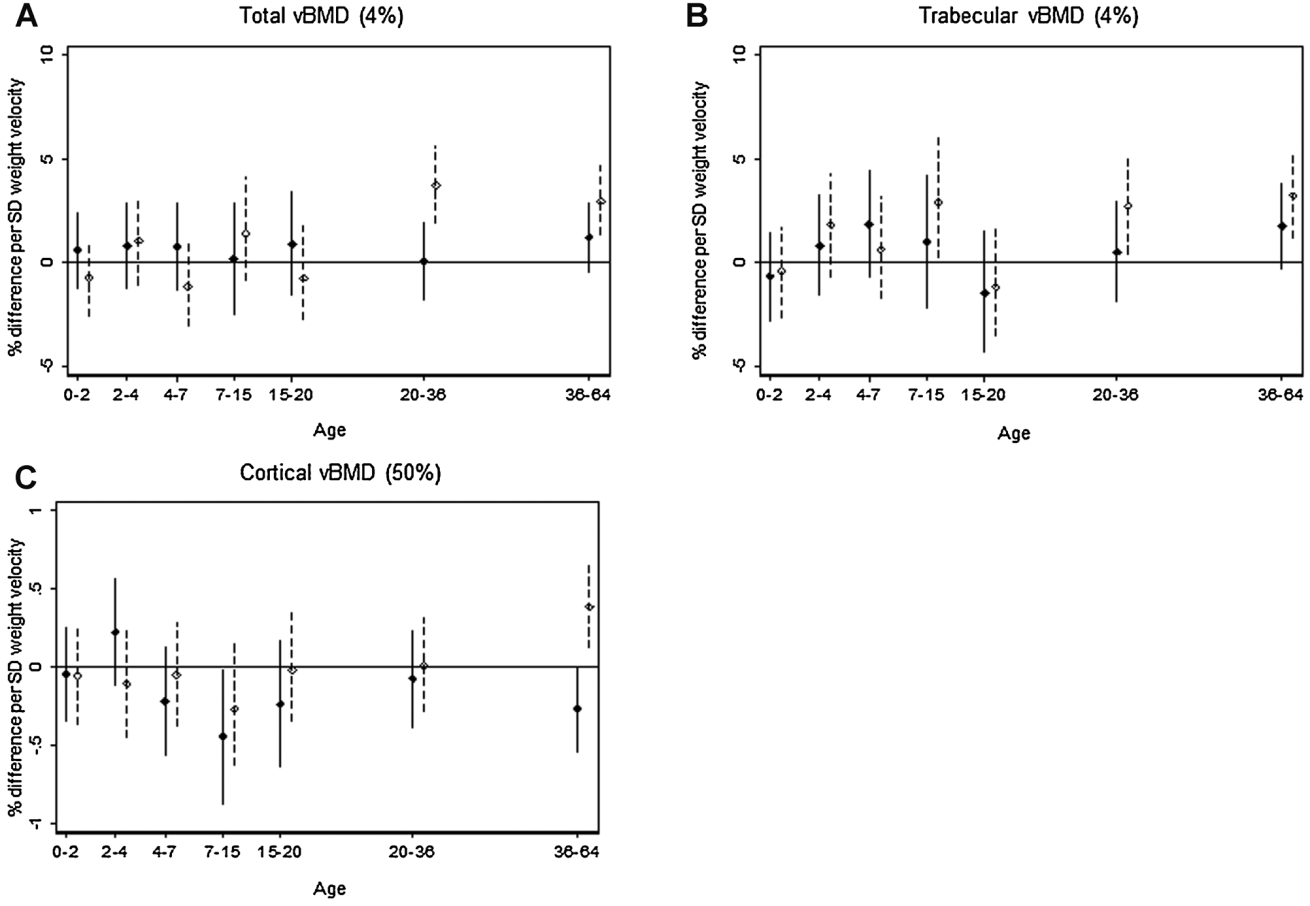
Weight velocity. Percentage difference and 95% CI in distal radius (*A*) total vBMD and (*B*) trabecular vBMD (4%), and (*C*) cortical vBMD (50%), at 60 to 64 years per 1 SD increase in weight velocity, adjusted for standardized height and weight at the beginning of the growth interval and corresponding standardized height velocity. Solid line for males; dashed line for females.

Height or weight gain velocity up to age 7 years was not associated with vBMD at either the diaphyseal or distal radius (Figs. 3 and 4, Supplementary Tables 3 and 4).

Greater height gain from 15 years in men was associated with lower distal radius trabecular vBMD (–5.0%; 95% CI, –8.2% to –2.0%) (Fig. 3*B*) and higher diaphyseal radius cortical vBMD (0.5%; 95% CI, 0.08% to 1.0%) (Fig. 3*C*). In women, greater height gain during 7 to 15 years was also associated with lower distal radius trabecular vBMD (–3.3%; 95% CI, –5.4% to –0.5%) (Fig. 3*B*).

In women, greater weight gain during 7 to 15 years was positively associated with trabecular vBMD (2.9%; 95% CI, 0.2% to 6.2%) (Fig. 4*B*), greater weight gain from 20 years with higher trabecular and total vBMD (eg, 3.7%; 95% CI, 1.9% to 5.7%) at 20 to 36 years (Fig. 4*A*), and greater weight gain from 36 years with higher cortical vBMD (0.4%; 95% CI, 0.1% to 0.7%) (Fig. 4*C*). For men, there was less evidence of an association with later weight gain with only weight gain 7 to 15 years being associated with lower diaphyseal radius cortical vBMD (Fig. 4*C*).

### Growth to adulthood and DXA measures of aBMD at the hip and lumbar spine at 60 to 64 years

Birth weight was not associated with aBMD at the hip or the lumbar spine, before or after adjusting for current height and weight (Table 2).

**Figure 5:**
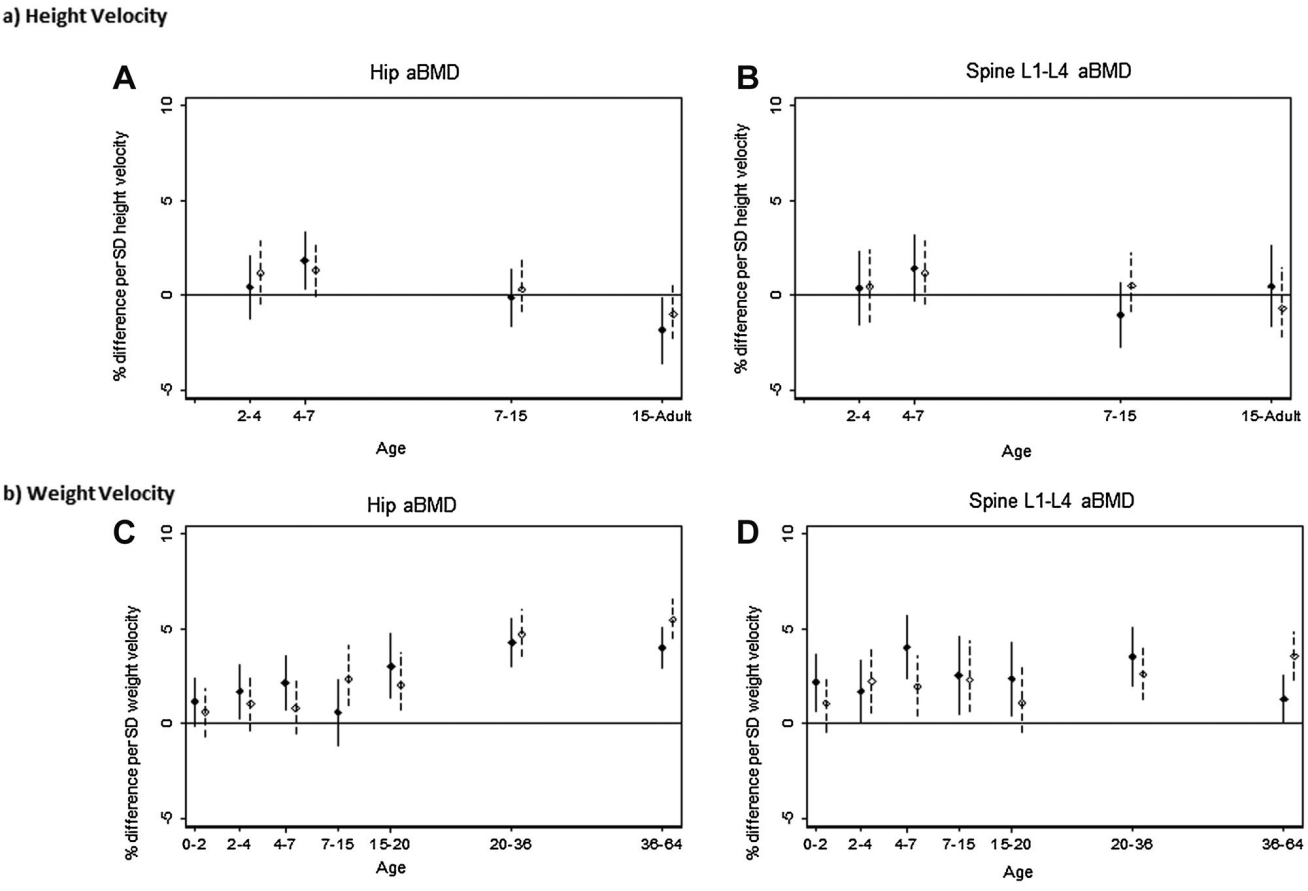
(*A*, *B*) Height velocity and (*C*, *D*) weight velocity. Percentage difference in aBMD at the hip and the lumbar spine at age 60 to 64 years per 1 SD increase in (*A*, *B*) height velocity or (*C*, *D*) weight velocity, adjusted for standardized height and weight at the beginning of the growth interval and corresponding standardized velocity. Solid line for males; dashed line for females.

Those who grew fastest in height between 4 and 7 years had greater hip aBMD at 60 to 64 years; for example, 1 SD increase in height velocity was associated with 1.8% (95% CI, 0.3% to 3.4%) difference in hip aBMD in men (Fig. 5*A*, Supplementary Table 5A). The patterns for women, and for lumbar spine aBMD (Fig. 5*B*) in both sexes, were similar but weaker. Associations with later height gain were either null or inverse.

Positive associations were observed between hip aBMD and weight gain from 2 to 4 years, 4 to 7 years, and from 15 years in men, and from 7 years in women (Fig. 5*C*, Supplementary Table 5B). The positive associations of weight gain in childhood and adolescence were more apparent for lumbar spine aBMD (Fig. 5*D*).

## Discussion

We have described associations between height and weight gain at different stages of growth and bone phenotype in a well-established birth cohort of adults aged 60 to 64 years at bone measurement. These analyses clearly demonstrate positive associations of birth weight and childhood and adolescent growth in height and weight with bone size, amount of cortical bone, and strength, with fewer and weaker associations with vBMD and aBMD. Adult weight gain was positively related to greater aBMD at the hip and lumbar spine, and greater vBMD at the radius in women. These findings may provide important information for fracture prevention interventions in later life.

### Birth weight and bone phenotype at 60 to 64 years

Birth weight was positively associated with diaphysis CSA, but not medullary CSA after adjustment for adult height and weight, indicating an association with the CSA and length of the skeletal envelope but not the amount of cortical bone within. Because SSI is an estimate of the torsional and bending strength of the bone, and combines vBMD and bone area, the positive association between birth weight and SSI is likely to be through greater bone CSA. These findings, together with the lack of positive associations between birth weight and cortical or trabecular vBMD and total hip or lumbar spine aBMD, agree with previous studies that have shown an association between birth weight and bone CSA, and between birth weight and strength in later life,^1^,^6^,^7^,^16^ and extend the knowledge base to a larger and younger cohort in early old age.

### Height gain and bone phenotype at 60 to 64 years

The importance of childhood height growth, particularly in the immediate prepubertal (4–7 years) and postpubertal (15 years to adult) periods, to differences in early old age in diaphyseal, medullary, distal CSA, and to SSI is indicated by the results of this study. Greater gains in height during these periods were associated with larger diaphysis CSA and may indicate appositional growth in response to the increase in the length of the bone, which would occur to maintain the integrity of bone to prevent skeletal fragility. This is consistent with longitudinal data in Canadian children in whom peak height velocity was followed by an increase in bone area.^17^ Prepubertal and postpubertal height growth were also associated with larger differences in medullary area in females than males, which suggests that males have greater cortical area per 1 SD gain in height compared to females. These differences may contribute to lower radius fracture risk in males compared to females.

At the 4% site, early (2–4 years) and postpubertal (15 years to adult) growth were related to distal CSA. There were no associations between height gain at any period with total vBMD at the distal radius at 60 to 64 years. Adult trabecular BMD was negatively associated with height gain at 7 to 15 years in females and at 15 years to adult in males. The negative association between height gain and trabecular vBMD may be related to biomechanical adaptation and redistribution of the bone toward periosteum in a larger bone, which would result in greater spacing between trabeculae and an apparent “decrease” in trabecular vBMD. This redistribution of bone would maintain strength while being light.^18^–^20^ In males, hip aBMD was also negatively associated with height gain postpuberty, again indicating redistribution of the bone as a biomechanical response to longitudinal growth.^19^

### Weight gain during childhood and adulthood and bone phenotype at 60 to 64 years

Infant weight gain was positively associated with increases in diaphysis and medullary CSA and consequently in SSI. Thereafter, weight gain at most stages of growth was positively related to diaphysis CSA and SSI. There were no periods of prepubertal weight gain associated with vBMD (cortical, total, or trabecular). In females, weight gain during adulthood was positively associated with total, trabecular, and cortical BMD, and with SSI, and was negatively associated with medullary CSA (indicating greater cortical area). Similarly, weight gain during most stages of growth and in the adult years was positively associated with hip aBMD and spine aBMD. These findings are consistent with greater weight velocity having similar effects on the radius and axial skeleton. The extent to which such weight gain will protect against fracture is unclear.^21^ However, it is well known that the maintenance of a healthy adult weight is important for bone health in later life.^22^ Any guidance would have to take into account that overweight and obesity are risk factors for many chronic diseases.

### Possible mechanisms

There are a number of possible interacting mechanisms (environmental, genetic, or epigenetic) that could drive the associations observed between higher birth weight, increased height, and weight gain during specific growth intervals, and also between greater adult diaphysis CSA, medullary CSA, and SSI. Mechanisms are likely to be different at different ages. Experimental studies demonstrate that alterations to the diet of pregnant animals can produce lasting changes in offspring bone physiology and structure,^23^ as well as affect birth weight. Epigenetic factors, such as alterations in DNA methylation, have also been established as mechanisms whereby the maternal environment can induce changes in gene expression in the developing fetus.^24^ After birth, adequate nutrition, particularly during periods of rapid height growth, is also extremely important for longitudinal and appositional growth as well as for healthy weight gain.^22^,^25^ Nutrition and exercise are likely to be the most important environmental modifiers for prepubertal skeletal growth. Positive associations observed during the later stages of growth may be due to the interactions between the sex hormones and the musculoskeletal system.^18^,^26^ The positive associations between greater height velocity in late puberty and bone geometry may reflect a later rise in or lowering of estradiol levels, because higher levels stop growth and inhibit periosteal expansion and endocortical resorption.^27^ Adipose and muscle tissue growth are likely to contribute to positive associations between weight gain and adult bone phenotype, either through biomechanical loading or via the effects of secretory products of the tissues. Consistent with this are previous findings from the NSHD cohort which showed that BMI gain in infancy and childhood was positively associated with greater adult lean mass^28^ and that prepubertal weight gain in males was associated with better physical performance, suggesting that early weight gain was associated with the development of lean muscle mass and function.^29^ It is also consistent with findings from the Helsinki cohort showing that thinness in childhood is a risk factor for adult hip fracture,^4^ and with findings from a Mendelian randomization study in the Avon Longitudinal Study of Parents and Children (ALSPAC) cohort, suggesting that fat mass is on the causal pathway for bone mass in children. Increased weight bearing and or increased estrogen or leptin levels may account for the positive associations observed in this study between adult weight gain and vBMD at the radius in women, and in aBMD at hip and lumbar spine in men and women.^30^–^32^ There are likely to be differential effects of any of the discussed mechanisms on the peripheral and axial skeleton, and at different stages of life.

### Strengths and limitations

This study has a number of strengths over previous studies. First, it investigated the whole growth trajectory and adult body size. Of the few previous studies with childhood growth measurements, only one^8^ had full growth data and adult body size; we added new evidence by having a population that had been followed for a longer time, into early old age. A second advantage was that both DXA and pQCT measurements were taken. Peripheral QCT allowed us to describe how growth trajectories were related to the amount of cortical bone, and the size and vBMD of bone, without confounding of body size. The third strength was the relatively large size of this British sample, which included men and women, and the narrow age range of the sample at assessment, which limited potential confounding by age.

One limitation of this study relates to the measurements available. Bone phenotype was only measured in early old age so we do not know whether the associations with various bone outcomes were the result of peak bone mass or adult bone loss. We could only characterize weight change rather than changes in lean and fat mass from body composition scans, as is now possible in younger cohorts.^33^,^34^ A second limitation is that we cannot translate our findings of the growth trajectory and future bone phenotype to fracture risk until sufficient events have accrued. However, our data are similar to those from the Helsinki cohort in which a low rate of childhood height gain was associated with future fracture risk,^2^ and that those in the lowest quartile of BMI gain, as a result of poor weight gain relative to height gain, were most likely to fracture.^5^ A third limitation of the study was that these analyses were limited to participants who attended a clinic visit. In our study, the clinic group was taller and the women were lighter than those who had only a home visit; and have also been found to have fewer health conditions.^35^ However, there is no reason to suspect that the associations between the growth parameters and the bone outcomes should differ between the two groups at this stage of early aging. A fourth limitation is that subjects in the sample were all born in the early postwar period. About 10% were stunted by age 6 years according to WHO growth charts, and few experienced excessive weight gain as children.^36^ Therefore, our findings may not be generalizable to later born cohorts who may be more likely to reach their height potential and who were exposed to the obesogenic environment at an earlier age. Rather, our findings may be more relevant for birth cohorts experiencing the transition from undernutrition to overnutrition, in which childhood underweight and stunting is more common.

## Conclusions

In a cohort born in the early postwar period, gaining weight and height faster than others—particularly through the prepubertal and postpubertal periods—and higher birth weight were positively related to bone strength, mostly through positive associations with bone CSA in early old age. Weight and height gain velocities are important at different stages of life and may explain differences in fracture risk between males and females. Moving forward, collection of fracture data in this cohort will help us ascertain how growth trajectories translate to fracture risk.
