# Supplementary figures and images for: Growth From Birth to Adulthood and Bone Phenotype in Early Old Age: A British Birth Cohort Study
Source: J Bone Miner Res. 2013 Dec 19;29(1):123–33. doi: 10.1002/jbmr.2008 (PMC4292430; doi:10.1002/jbmr.2008)

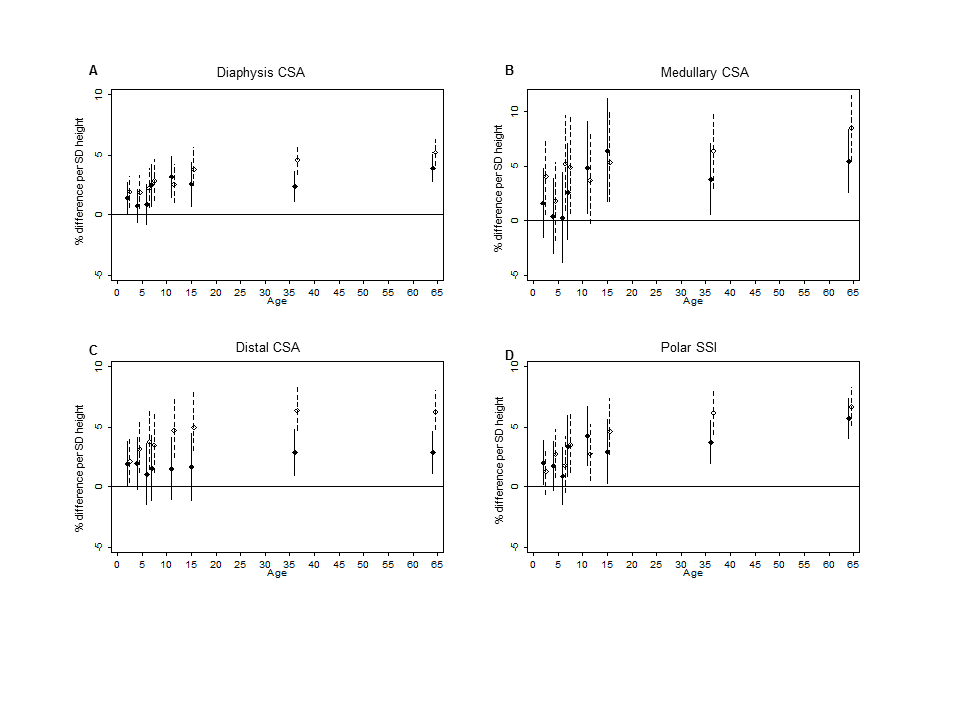

Supplement: Supplementary file 1 [file jbmr0029-0123-sd1.tif]

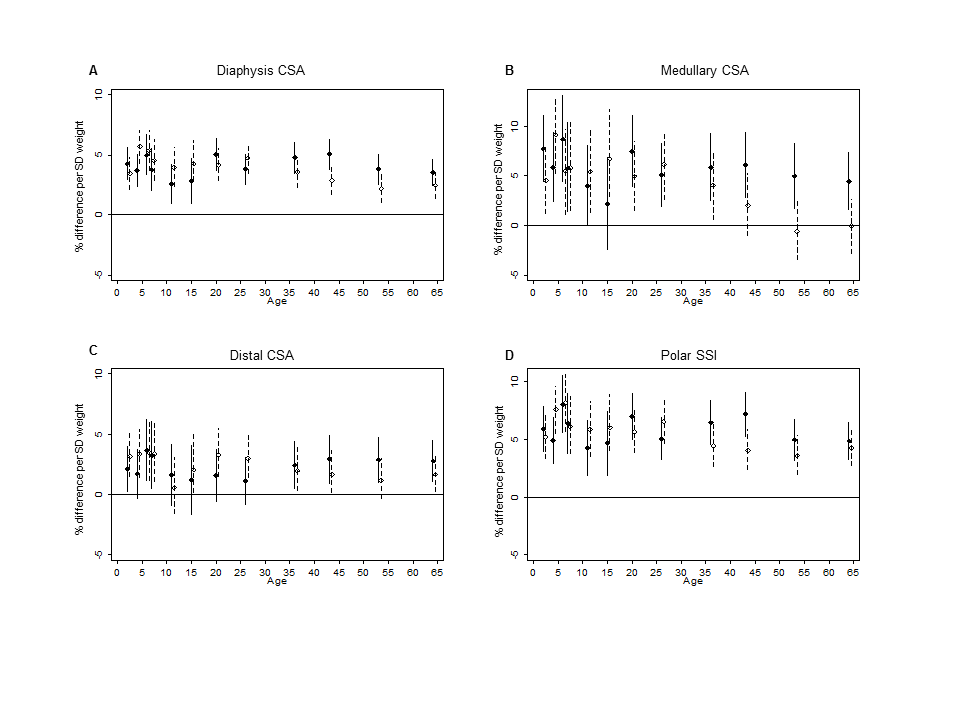

Supplement: Supplementary file 2 [file jbmr0029-0123-sd2.tif]

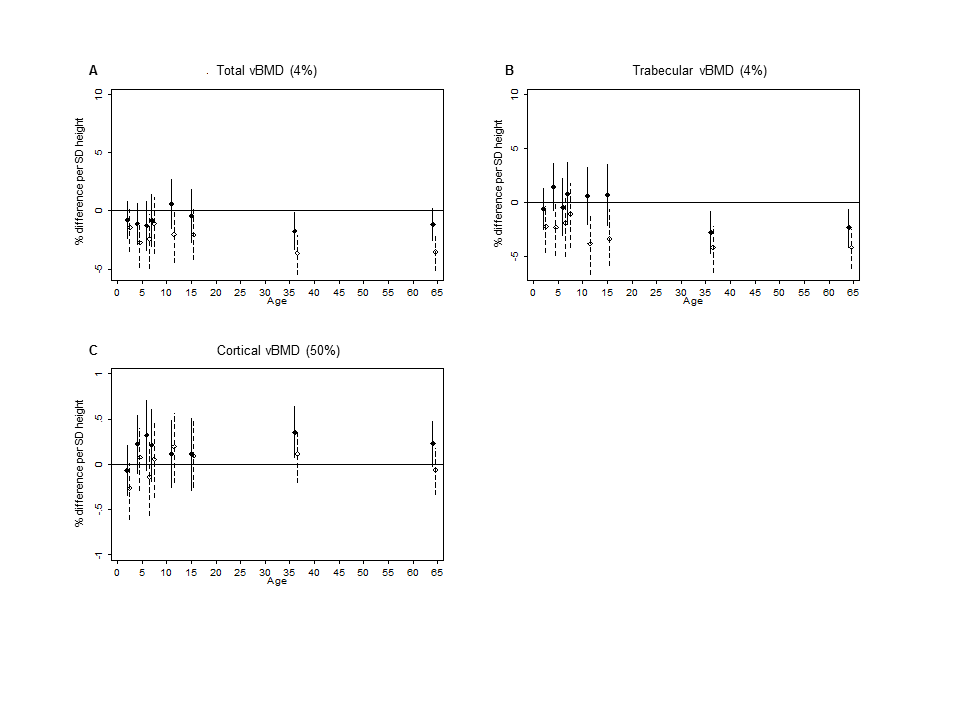

Supplement: Supplementary file 3 [file jbmr0029-0123-sd3.tif]

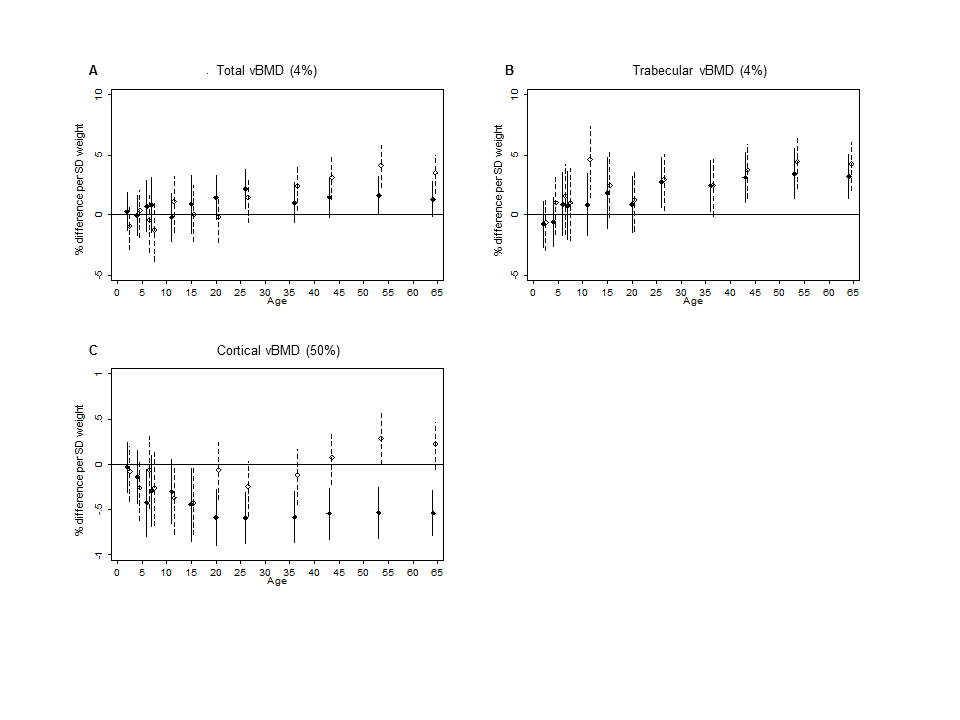

Supplement: Supplementary file 4 [file jbmr0029-0123-sd4.tif]

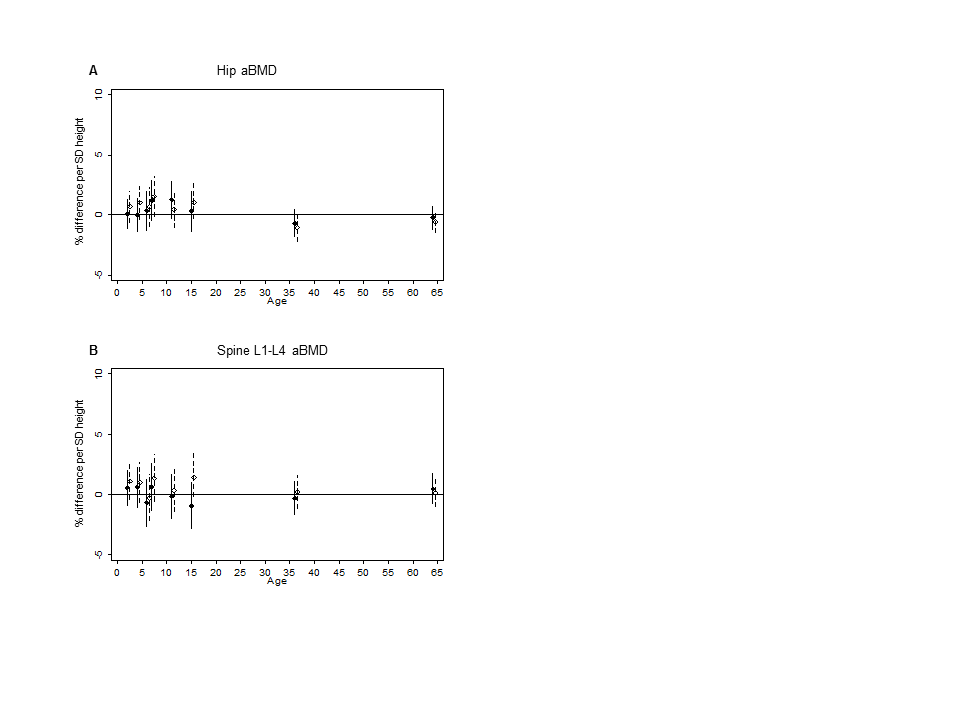

Supplement: Supplementary file 5 [file jbmr0029-0123-sd5.tif]

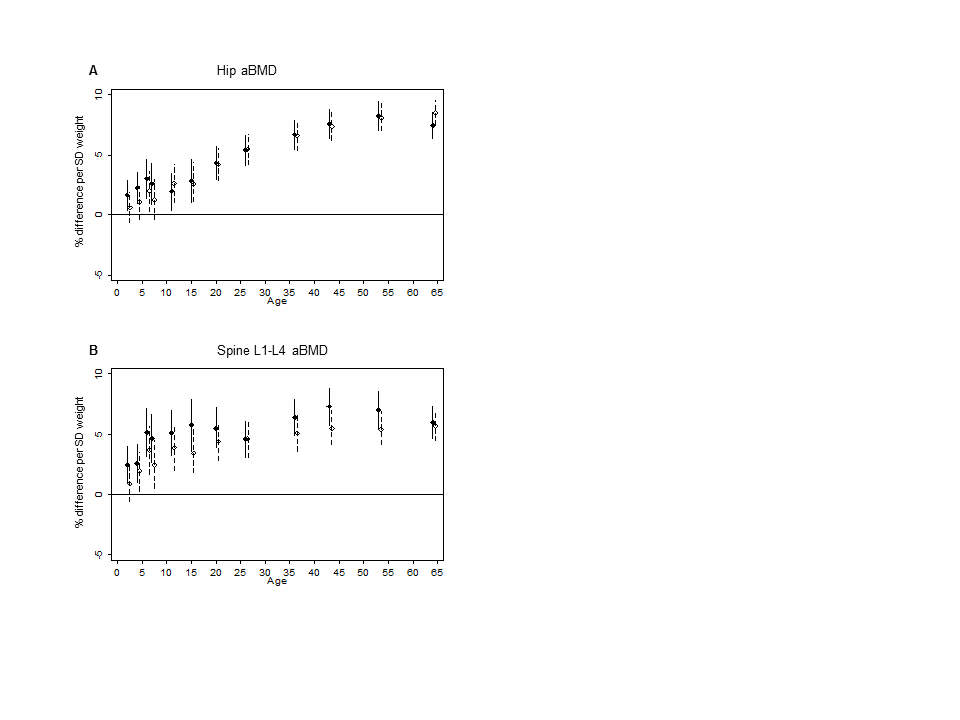

Supplement: Supplementary file 6 [file jbmr0029-0123-sd6.tif]
