# Supplementary material for: Growth From Birth to Adulthood and Bone Phenotype in Early Old Age: A British Birth Cohort Study
Source: J Bone Miner Res. 2013 Dec 19;29(1):123–33. doi: 10.1002/jbmr.2008 (PMC4292430; doi:10.1002/jbmr.2008)
Supplement: Supplementary file 7 [file jbmr0029-0123-sd7.docx]

**Supplementary Tables 1-5**

**Supplementary Table 1**. Height velocity. Percentage difference and 95% CI in radius diaphysis and medullary CSA, polar SSI (50% site) and distal CSA (4% site at 60-64 years per one standard deviation increase in height velocity adjusted for standardised height and weight at the beginning of the growth interval and corresponding standardised weight velocity. N values range from 933-1025.

|  | Diaphysis CSA (50%) | | Medullary CSA (50%) | | Distal CSA (4%) | | Polar SSI | |
| --- | --- | --- | --- | --- | --- | --- | --- | --- |
|  | MEN  % difference  (95% CI) | WOMEN  % difference  (95% CI) | MEN  % difference  (95% CI) | WOMEN  % difference  (95% CI) | MEN  % difference  (95% CI) | WOMEN  % difference  (95% CI) | MEN  % difference  (95% CI) | WOMEN  % difference  (95% CI) |
| Height velocity |  |  |  |  |  |  |  |  |
| 2-4 years (SD) | 0.96  (-0.68, 2.63) | 1.68  (0.11, 3.37) | -0.34  (4.45, 3.99) | 1.07  (-2.94, 5.46) | 2.62  (0.27, 5.02) | 2.45  (0.20, 4.85) | 2.62  (0.25, 5.04) | 2.79  (0.53, 5.22) |
| 4-7 years (SD) | 2.38  (0.83, 3.95) | 2.14  (0.76, 3.71) | 2.95  (-1.05, 7.11) | 4.67  (1.03, 8.90) | 0.75  (-1.48, 3.04) | 1.71  (-0.34, 4.01) | 3.33  (1.13, 5.58) | 2.56  (0.61, 4.79) |
| 7-15 years (SD) | 0.45  (-1.13, 2.06) | 1.18  (-0.06, 2.80) | 2.66  (-1.39, 6.88) | 1.40  (-1.73, 5.57) | 0.50  (-1.81, 2.86) | 2.70  (0.85, 5.11) | 0.34  (-1.84, 2.57) | 1.80  (0.07, 4.06) |
| 15-Adult (SD) | 1.76  (-0.13, 3.68) | 3.46  (2.08, 5.42) | 1.91  (-2.89, 6.95) | 5.30  (1.72, 10.51) | 3.33  (0.62, 6.10) | 3.69  (1.74, 6.48) | 3.02  (0.35, 5.75) | 3.99  (2.06, 6.75) |

**Supplementary Table 2**. Weight velocity, Percentage difference and 95% CI in radius diaphysis and medullary CSA, polar SSI (50% site) and distal CSA (4% site) at 60-64 years per one standard deviation increase in weight velocity adjusted for standardised height and weight at the beginning of the growth interval and corresponding standardised height velocity. N values range from 912-1232.

|  | Diaphysis CSA (50%) | | Medullary CSA (50%) | | Distal CSA (4%) | | Polar SSI | |
| --- | --- | --- | --- | --- | --- | --- | --- | --- |
|  | MEN  % difference  (95% CI) | WOMEN  % difference  (95% CI) | MEN  % difference  (95% CI) | WOMEN  % difference  (95% CI) | MEN  % difference  (95% CI) | WOMEN  % difference  (95% CI) | MEN  % difference  (95% CI) | WOMEN  % difference  (95% CI) |
| Weight velocity |  |  |  |  |  |  |  |  |
| 0-2 years (SD) | 3.42  (2.12, 4.74) | 2.69  (1.37, 4.00) | 6.82  (3.47, 10.28) | 3.87  (0.57, 7.23) | 1.10  (-0.65, 2.87) | 2.41  (0.59, 4.21) | 4.71  (2.83, 6.63) | 4.04  (2.15, 5.94) |
| 2-4 years (SD) | 1.30  (-0.09, 2.71) | 3.75  (2.30, 5.20) | 0.93  (-2.62, 4.60) | 5.61  (1.81, 9.45) | 0.42  (-1.51, 2.38) | 1.25  (-0.76, 3.23) | 1.80  (-0.17, 3.82) | 5.04  (2.95, 7.12) |
| 4-7 years (SD) | 2.07  (0.68, 3.49) | 1.70  (0.38, 3.11) | 2.39  (-1.21, 6.13) | 2.04  (-1.38, 5.76) | 3.21  (1.11, 5.34) | 1.32  (-0.64, 3.42) | 4.15  (2.14, 6.19) | 2.23  (0.36, 4.24) |
| 7-15 years (SD) | 0.58  (-1.21, 2.41) | 1.54  (0.05, 3.38) | -0.33  (-4.78, 4.33) | 2.61  (-1.16, 7.41) | -1.54  (-4.09, 1.08) | -0.46  (-2.59, 2.18) | 0.42  (-2.05, 2.96) | 2.04  (0.03, 4.62) |
| 15-20 years (SD) | 3.74  (2.06, 5.44) | 1.53  (0.21, 3.19) | 6.21  (1.80, 10.81) | 3.18  (-0.29, 7.64) | 0.62  (-1.79, 3.09) | 1.73  (-0.26, 4.22) | 4.66  (2.28, 7.09) | 1.41  (-0.45, 3.76) |
| 20-36 years (SD) | 1.17  (-0.11, 2.47) | 0.88  (-0.30, 2.17) | -0.36  (-3.61, 3.00) | -0.84  (-3.81, 2.50) | 2.43  (0.59, 4.31) | -0.24  (-1.90, 1.59) | 2.01  (0.19, 3.87) | 1.11  (-0.55, 2.95) |
| 36-64 years (SD) | 0.38  (-0.75, 1.52) | 0.28  (-0.79, 1.42) | 0.43  (-2.49, 3.43) | -3.21  (-5.87, -0.32) | 1.65  (0.06, 3.26) | 0.29  (-1.21, 1.88) | 0.32  (-1.26, 1.94) | 1.85  (0.33, 3.49) |

Table 3. Height velocity. Percentage difference and 95% CI in distal radius (4%), total and trabecular vBMD, and cortical vBMD (50%) at 60-64 years per one standard deviation increase in height velocity adjusted for standardised height and weight at the beginning of the growth interval and corresponding standardised weight velocity. N values range from 933-1025.

|  | Total vBMD (4%) | | Trabecular vBMD (4%) | | Cortical vBMD (50%) | |
| --- | --- | --- | --- | --- | --- | --- |
|  | MEN  % difference  (95% CI) | WOMEN  % difference  (95% CI) | MEN  % difference  (95% CI) | WOMEN  % difference  (95% CI) | MEN  % difference  (95% CI) | WOMEN  % difference  (95% CI) |
| Height velocity |  |  |  |  |  |  |
| 2-4 years (SD) | -1.69  (-4.06, 0.74) | -1.83  (-4.09, 0.60) | 1.91  (-1.00, 4.91) | -0.98  (-3.69, 1.93) | 0.28  (-0.13, 0.69) | 0.11  (-0.28, 0.52) |
| 4-7 years (SD) | 0.38  (-1.90, 2.71) | 0.47  (-1.60, 2.81) | 2.06  (-0.73, 4.92) | 1.56  (-0.96, 4.41) | 0.03  (-0.36, 0.41) | -0.05  (-0.39, 0.34) |
| 7-15 years (SD) | 0.55  (-1.82, 2.98) | -1.47  (-3.29, 0.91) | 0.36  (-2.42, 3.23) | -3.31  (-5.41,-0.54) | 0.11  (-0.28, 0.50) | 0.11  (-0.19, 0.50) |
| 15-Adult (SD) | -2.44  (-5.15, 0.36) | -2.42  (-4.38, 0.37) | -5.01  (-8.16, -1.74) | -2.02  (-4.37, 1.34) | 0.54  (0.08, 0.99) | -0.13  (-0.46, 0.32) |

Table 4. Weight velocity. Percentage difference and 95% CI in distal radius (4%), total and trabecular vBMD, and cortical vBMD (50%) at 60-64 years per one standard deviation increase in weight velocity adjusted for standardised height and weight at the beginning of the growth interval and corresponding standardised height velocity. N values range from 912-1232

|  | Total vBMD (4%) | | Trabecular vBMD (4%) | | Cortical vBMD (50%) | |
| --- | --- | --- | --- | --- | --- | --- |
|  | MEN  % difference  (95% CI) | WOMEN  % difference  (95% CI) | MEN  % difference  (95% CI) | WOMEN  % difference  (95% CI) | MEN  % difference  (95% CI) | WOMEN  % difference  (95% CI) |
| Weight velocity |  |  |  |  |  |  |
| 0-2 years (SD) | 0.61  (-1.20, 2.45) | -0.73  (-2.56, 1.09) | -0.67  (-2.80, 1.50) | -0.42  (-2.62, 1.76) | -0.04  (-0.35, 0.26) | -0.06  (-0.37, 0.25) |
| 2-4 years (SD) | 0.81  (-1.23, 2.88) | 1.06  (-1.05, 3.14) | 0.82  (-1.60, 3.29) | 1.82  (-0.70, 4.32) | 0.22  (-0.12, 0.57) | -0.10  (-0.46, 0.24) |
| 4-7 years (SD) | 0.78  (-1.31, 2.92) | -1.15  (-3.11, 0.95) | 1.86  (-0.69, 4.47) | 0.64  (-1.77, 3.22) | -0.21  (-0.56, 0.13) | -0.05  (-0.38, 0.29) |
| 7-15 years (SD) | 0.20  (-2.47, 2.94) | 1.39  (-0.84, 4.17) | 1.00  (-2.16, 4.27) | 2.89  (0.23, 6.22) | -0.44  (-0.88, -0.01) | -0.27  (-0.63, 0.17) |
| 15-20 years (SD) | 0.90  (-1.61, 3.47) | -0.73  (-2.74, 1.80) | -1.44  (-4.35, 1.56) | -1.18  (-3.56, 1.83) | -0.24  (-0.64, 0.17) | -0.02  (-0.35, 0.39) |
| 20-36 years (SD) | 0.08  (-1.81, 2.00) | 3.72  (1.91, 5.71) | 0.52  (-1.89, 2.98) | 2.72  (0.44, 5.24) | -0.07  (-0.39, 0.24) | 0.01  (-0.28, 0.33) |
| 36-64 years (SD) | 1.23  (-0.44, 2.93) | 2.96  (1.33, 4.69) | 1.76  (-0.33, 3.89) | 3.22  (1.18, 5.38) | -0.26  (-0.54, 0.01) | 0.38  (0.12, 0.66) |

Table 5 a) Height velocity and b) Weight velocity**.** Percentage difference in aBMD at the hip and the lumbar spine at 60-64 years per one standard deviation increase in a) height velocity or b) weight velocity adjusted for standardised height and weight at the beginning of the growth interval and corresponding standardised velocity. N values range from 1106-1494

|  | Hip aBMD | | Spine L1-L4 aBMD | |
| --- | --- | --- | --- | --- |
|  | MEN  % difference  (95% CI) | WOMEN  % difference  (95% CI) | MEN  % difference  (95% CI) | WOMEN  % difference  (95% CI) |
| a) Height velocity |  |  |  |  |
| 2-4 years (SD) | 0.44  (-1.26, 2.17) | 1.18  (-0.44, 2.92) | 0.38  (-1.58, 2.38) | 0.46  (-1.40, 2.46) |
| 4-7 years (SD) | 1.83  (0.29, 3.39) | 1.33  (-0.08, 2.88) | 1.42  (-0.32, 3.19) | 1.16  (-0.45, 2.92) |
| 7-15 years (SD) | -0.11  (-1.64, 1.44) | 0.32  (-0.88, 1.88) | -1.05  (-2.76, 0.70) | 0.51  (-0.87, 2.28) |
| 15-Adult (SD) | -1.82  (-3.57, -0.02) | -1.01  (-2.29, 0.80) | 0.50  (-1.63, 2.68) | -0.69  (-2.22, 1.46) |
|  |  |  |  |  |
| b) Weight velocity |  |  |  |  |
| 0-2 years (SD) | 1.15  (-0.16, 2.47) | 0.62  (-0.66, 1.94) | 2.17  (0.65, 3.72) | 1.08  (-0.42, 2.60) |
| 2-4 years (SD) | 1.70  (0.25, 3.18) | 1.04  (-0.41, 2.51) | 1.71  (0.05, 3.39) | 2.25  (0.55, 3.94) |
| 4-7 years (SD) | 2.15  (0.70, 3.62) | 0.82  (-0.53, 2.28) | 4.02  (2.34, 5.72) | 1.96  (0.40, 3.63) |
| 7-15 years (SD) | 0.60  (-1.18, 2.41) | 2.39  (0.93, 4.23) | 2.55  (0.49, 4.66) | 2.31  (0.65, 4.41) |
| 15-20 years (SD) | 3.05  (1.33, 4.79) | 2.04  (0.68, 3.76) | 2.36  (0.41, 4.35) | 1.09  (-0.44, 3.06) |
| 20-36 years (SD) | 4.28  (2.99, 5.58) | 4.72  (3.54, 6.03) | 3.54  (2.00, 5.11) | 2.62  (1.23, 4.17) |
| 36-64 years (SD) | 4.02  (2.96, 5.58) | 5.50  (4.51, 6.59) | 1.31  (0.01, 2.63) | 3.54  (2.31, 4.89) |
